# *anti*-Tricyclo­[4.2.1.1^2,5^]deca-3,7-diene-9-*endo*,10*-endo*-diol

**DOI:** 10.1107/S1600536808035423

**Published:** 2008-11-08

**Authors:** Andria D. Harris, Amy D. Baucom, Maria del Rosario I. Amado Sierra, Daniel S. Jones, Markus Etzkorn

**Affiliations:** a Department of Chemistry, The University of North Carolina at Charlotte, 9201 University City Boulevard, Charlotte, NC 28223, USA

## Abstract

The title compound, C_10_H_12_O_2_, was synthesized as a candidate for further functionalization. The asymmetric unit comprises two independent mol­ecules, both of which are situated on a center of symmetry. Both mol­ecules are involved in a network of hydrogen bonding, with each alcohol group participating in one hydrogen bond as a donor and in a second hydrogen bond as an acceptor.

## Related literature

For a related structure, see: Eaton *et al.* (2002 ▶). For synthesis details, see: Baggiolini *et al.* (1967 ▶); Klinsmann *et al.* (1972 ▶); Prakash *et al.* (1987 ▶); Herzog (1958 ▶). For synthesis details and compound characterization, see: Amman *et al.* (1980 ▶). For synthetic routes utilizing the title compound as a starting material, see: Amman & Ganter (1977 ▶, 1981 ▶).
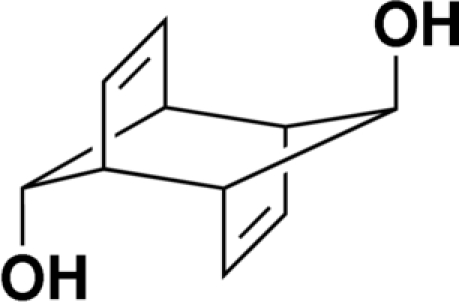


## Experimental

### 

#### Crystal data

C_10_H_12_O_2_
*M* *_r_* = 164.2
Monoclinic, 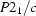

*a* = 10.3730 (14) Å
*b* = 9.8494 (14) Å
*c* = 7.7128 (11) Å
β = 91.850 (11)°
*V* = 787.59 (19) Å^3^
*Z* = 4
Cu *K*α radiation
μ = 0.77 mm^−1^
*T* = 295 (2) K
0.5 × 0.5 × 0.5 mm

#### Data collection

Enraf–Nonius CAD-4 diffractometer
Absorption correction: none
5405 measured reflections
1419 independent reflections
1386 reflections with *I* > 2σ(*I*)
*R*_int_ = 0.038
3 standard reflections frequency: 60 min intensity decay: none

#### Refinement

*R*[*F* ^2^ > 2σ(*F* ^2^)] = 0.039
*wR*(*F* ^2^) = 0.100
*S* = 1.08
1419 reflections
112 parameters
H-atom parameters constrained
Δρ_max_ = 0.25 e Å^−3^
Δρ_min_ = −0.19 e Å^−3^

### 

Data collection: *CAD-4 EXPRESS* (Enraf–Nonius, 1994 ▶); cell refinement: *CAD-4 EXPRESS*; data reduction: *XCAD4* (Harms & Wocadlo, 1995 ▶); program(s) used to solve structure: *SHELXS97* (Sheldrick, 2008 ▶); program(s) used to refine structure: *SHELXL97* (Sheldrick, 2008 ▶); molecular graphics: *ORTEP-3 for Windows* (Farrugia, 1997 ▶); software used to prepare material for publication: *WinGX* (Farrugia, 1999 ▶).

## Supplementary Material

Crystal structure: contains datablocks global, I. DOI: 10.1107/S1600536808035423/fj2159sup1.cif

Structure factors: contains datablocks I. DOI: 10.1107/S1600536808035423/fj2159Isup2.hkl

Additional supplementary materials:  crystallographic information; 3D view; checkCIF report

## Figures and Tables

**Table 1:** Hydrogen-bond geometry (Å, °)

*D*—H⋯*A*	*D*—H	H⋯*A*	*D*⋯*A*	*D*—H⋯*A*
O1—HO1⋯O2^i^	0.82	1.95	2.7452 (15)	163
O2—HO2⋯O1	0.82	1.99	2.8005 (14)	170

Symmetry code: (i) 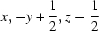.

**Fig. 1.**
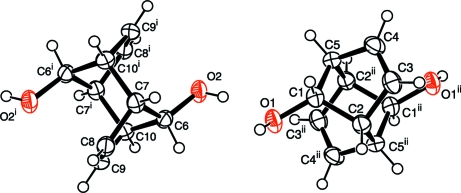
View of the two independent molecules of the title compound, 4, with 50% probability displacement ellipsoids. [Symmetry codes: (i) -x + 1, -y, -z; (ii) -x + 2, -y + 1, -z]

**Fig. 2.**
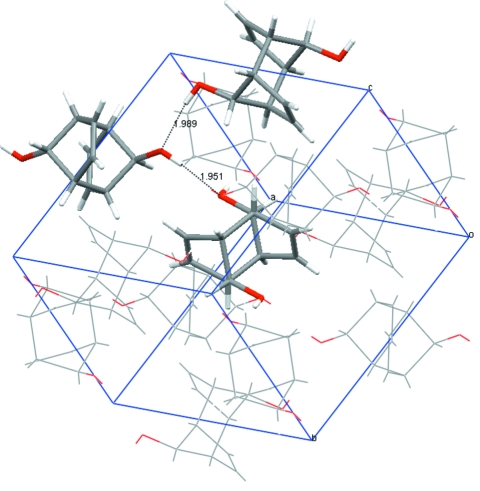
Packing diagram showing two of the hydrogen bonds in the hydrogen bonding network of the structure.

**Fig. 3.**
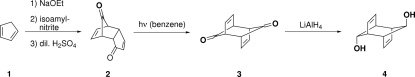
The formation of the title compound.

## supplementary crystallographic information

### Comment

The polycyclic title compound 4 has gained importance as a precursor to a bishomoaromatic dication (Prakash *et al.*, 1987) and was furthermore investigated in a synthetic approach to heterodiamantanes (Amman *et al.*, 1980; Amman & Ganter, 1977; Amman & Ganter, 1981). We were interested in the functionalization of dienedione 3, which is a rather challenging task if one considers the issue of chemoselectivity, regioselectivity and stereoselectivity in a relatively small polycyclic skeleton with two olefinic bonds and two carbonyl groups in close proximity. Furthermore, a thermally induced isomerization of compound 3 to the *endo-*cyclopentadienone dimer 2 (Baggiolini *et al.*, 1967; Klinsmann *et al.*, 1972) prohibited several functionalization reactions that required forcing conditions. We therefore focused on the thermally stable dienediol 4.

Two pairs of independent molecules (Figure 1) comprise the four molecules in the unit cell, each of which is situated on a center of symmetry. All of the molecules are involved in a network of hydrogen bonding (Figure 2), with each alcohol group participating in one hydrogen bond as a donor and in a second hydrogen bond as an acceptor.

### Experimental

*Preparation of dienedione 2 *(Herzog, 1958): A solution of sodium ethoxide in ethanol was prepared by adding sodium (44.4 g, 1.94 mol) in small pieces to ethanol (2 l). A mixture of freshly distilled cyclopentadiene monomer (74 g, 1.12 mol) and isoamylnitrite (131 g, 1.12 mol) was then added dropwise at 15–20 °C. The dark brown reaction mixture was stirred for 30 minutes, poured on ice (0.5 kg) and extracted with ether (3 × 300 ml). These organic extracts were discarded, and the aqueous phase was acidified to pH 3 with 2.5 *M* sulfuric acid (*ca* 600 ml). After saturating with sodium chloride (*ca* 250 g) the aqueous phase was extracted with ether (5 × 600 ml). The combined organic phase was concentrated to a third of its volume, washed with water (100 ml) and dried (MgSO_4_). After removal of the solvent the viscous brown oil obtained (62 g) was refluxed in 1*M* sulfuric acid (1500 ml) for several hours and stirred at room temperature overnight. The reaction mixture was saturated with sodium chloride (*ca* 0.5 kg) and extracted with ether (5 × 400 ml). The combined organic phase was concentrated, washed with water, and dried (MgSO_4_). After removal of the solvent a pale-brown crude product was obtained. Sublimation furnished the clean product (27 g, 30% with respect to cyclopentadiene) as a colorless solid.

After the photoisomerization of dienedione 2 to dienedione 3 (Baggiolini *et al., *1967; Klinsmann *et al.*, 1972) a reduction with LiAlH_4 _in THF yielded diol 4 (Amman *et al.*, 1980; Prakash *et al.*, 1987). We were able to grow single crystals of diol 4 from CHCl_3 _and thus provide structural details of the otherwise fully characterized compound (Amman *et al.*, 1980).

### Refinement

H atoms were constrained using a riding model. The olefinic C—H bond lengths were fixed at 0.93 Å and the methine C—H bond lengths at 0.98 Å, with *U*_iso_(H) = 1.2 U_eq._ (C). The O—H bond lengths were fixed at 0.82 Å, with *U*_iso_(H) = 1.5 U_eq._ (C), and the torsion angles about the C—O bonds were refined.

### Crystal data

**Table:**

C_10_H_12_O_2_	*F*_000_ = 352
*M**_r_* = 164.2	*D*_x_ = 1.385 Mg m^−^^3^
Monoclinic, *P*2_1_/*c*	Cu *K*α radiation λ = 1.54184 Å
Hall symbol: -P 2ybc	Cell parameters from 22 reflections
*a* = 10.3730 (14) Å	θ = 36.5–41.8º
*b* = 9.8494 (14) Å	µ = 0.77 mm^−^^1^
*c* = 7.7128 (11) Å	*T* = 295 (2) K
β = 91.850 (11)º	Irregular, brown
*V* = 787.59 (19) Å^3^	0.5 × 0.5 × 0.5 mm
*Z* = 4	

### Data collection

**Table:**

Enraf–Nonius CAD-4 diffractometer	θ_min_ = 4.3º
Nonprofiled ω/2θ scans	*h* = −12→12
Absorption correction: none	*k* = −11→11
5405 measured reflections	*l* = −9→9
1419 independent reflections	3 standard reflections
1386 reflections with *I* > 2σ(*I*)	every 60 min
*R*_int_ = 0.038	intensity decay: none
θ_max_ = 67.5º	

### Refinement

**Table:**

Refinement on *F*^2^	H-atom parameters constrained
Least-squares matrix: full	*w* = 1/[σ^2^(*F*_o_^2^) + (0.0449*P*)^2^ + 0.2879*P*] where *P* = (*F*_o_^2^ + 2*F*_c_^2^)/3
*R*[*F*^2^ > 2σ(*F*^2^)] = 0.039	(Δ/σ)_max_ < 0.001
*wR*(*F*^2^) = 0.100	Δρ_max_ = 0.25 e Å^−^^3^
*S* = 1.08	Δρ_min_ = −0.18 e Å^−^^3^
1419 reflections	Extinction correction: SHELXL97 (Sheldrick, 2008), Fc^*^=kFc[1+0.001xFc^2^λ^3^/sin(2θ)]^-1/4^
112 parameters	Extinction coefficient: 0.025 (2)

### Special details

**Table:**

Geometry. All e.s.d.'s (except the e.s.d. in the dihedral angle between two l.s. planes) are estimated using the full covariance matrix. The cell e.s.d.'s are taken into account individually in the estimation of e.s.d.'s in distances, angles and torsion angles; correlations between e.s.d.'s in cell parameters are only used when they are defined by crystal symmetry. An approximate (isotropic) treatment of cell e.s.d.'s is used for estimating e.s.d.'s involving l.s. planes.

### Fractional atomic coordinates and isotropic or equivalent isotropic displacement parameters (Å^2^)

**Table:**

	*x*	*y*	*z*	*U*_iso_*/*U*_eq_	
O1	0.79276 (9)	0.37798 (11)	−0.08942 (13)	0.0372 (3)	
HO1	0.7463	0.3684	−0.1766	0.056*	
O2	0.68564 (9)	0.15711 (10)	0.08353 (13)	0.0375 (3)	
HO2	0.7252	0.2197	0.0396	0.056*	
C10	0.44926 (13)	0.09207 (14)	0.11758 (19)	0.0306 (3)	
H10	0.4357	0.1453	0.2228	0.037*	
C2	0.96268 (12)	0.55937 (14)	−0.15964 (18)	0.0301 (3)	
H2	0.9118	0.607	−0.2501	0.036*	
C7	0.53244 (12)	0.06358 (14)	−0.15675 (17)	0.0296 (3)	
H7	0.5816	0.0942	−0.2559	0.036*	
C5	1.03469 (12)	0.36341 (14)	−0.01805 (19)	0.0309 (3)	
H5	1.0383	0.2646	−0.0035	0.037*	
C1	0.92078 (12)	0.40891 (14)	−0.13877 (18)	0.0307 (3)	
H1	0.9328	0.3648	−0.251	0.037*	
C6	0.55870 (13)	0.15044 (13)	0.00772 (18)	0.0295 (3)	
H6	0.5339	0.2437	−0.0224	0.035*	
C3	1.09864 (13)	0.52746 (16)	−0.21451 (18)	0.0352 (4)	
H3	1.1454	0.5778	−0.2928	0.042*	
C8	0.39004 (13)	0.09802 (14)	−0.1751 (2)	0.0342 (4)	
H8	0.3435	0.1069	−0.2796	0.041*	
C9	0.34268 (13)	0.11333 (14)	−0.0195 (2)	0.0352 (4)	
H9	0.2573	0.1339	0.0027	0.042*	
C4	1.13997 (13)	0.41653 (15)	−0.1333 (2)	0.0363 (4)	
H4	1.2207	0.3772	−0.1448	0.044*	

### Atomic displacement parameters (Å^2^)

**Table:**

	*U*^11^	*U*^22^	*U*^33^	*U*^12^	*U*^13^	*U*^23^
O1	0.0281 (5)	0.0435 (6)	0.0395 (6)	−0.0110 (4)	−0.0058 (4)	0.0054 (5)
O2	0.0326 (6)	0.0343 (6)	0.0447 (6)	−0.0107 (4)	−0.0120 (4)	0.0087 (4)
C10	0.0302 (7)	0.0286 (7)	0.0332 (7)	0.0013 (5)	0.0017 (5)	−0.0035 (5)
C2	0.0264 (7)	0.0336 (7)	0.0301 (7)	−0.0008 (5)	−0.0023 (5)	0.0079 (6)
C7	0.0291 (7)	0.0315 (7)	0.0281 (7)	−0.0037 (5)	−0.0017 (5)	0.0057 (5)
C5	0.0278 (7)	0.0246 (6)	0.0403 (8)	0.0030 (5)	0.0023 (6)	0.0017 (6)
C1	0.0276 (7)	0.0332 (7)	0.0312 (7)	−0.0041 (5)	0.0004 (5)	−0.0012 (6)
C6	0.0285 (7)	0.0241 (6)	0.0353 (8)	−0.0024 (5)	−0.0060 (5)	0.0037 (5)
C3	0.0311 (7)	0.0438 (8)	0.0310 (7)	−0.0054 (6)	0.0067 (6)	0.0003 (6)
C8	0.0313 (7)	0.0279 (7)	0.0424 (8)	−0.0039 (6)	−0.0124 (6)	0.0100 (6)
C9	0.0253 (7)	0.0271 (7)	0.0530 (9)	0.0041 (5)	−0.0012 (6)	0.0042 (6)
C4	0.0278 (7)	0.0405 (8)	0.0411 (8)	0.0040 (6)	0.0075 (6)	−0.0050 (6)

### Geometric parameters (Å, °)

**Table:**

O1—C1	1.4261 (16)	C7—H7	0.98
O1—HO1	0.82	C5—C4	1.5227 (18)
O2—C6	1.4248 (16)	C5—C1	1.5469 (19)
O2—HO2	0.82	C5—C2^ii^	1.567 (2)
C10—C9	1.520 (2)	C5—H5	0.98
C10—C6	1.5490 (18)	C1—H1	0.98
C10—C7^i^	1.5728 (18)	C6—H6	0.98
C10—H10	0.98	C3—C4	1.324 (2)
C2—C3	1.5183 (18)	C3—H3	0.93
C2—C1	1.5542 (19)	C8—C9	1.320 (2)
C2—C5^ii^	1.567 (2)	C8—H8	0.93
C2—H2	0.98	C9—H9	0.93
C7—C8	1.5178 (18)	C4—H4	0.93
C7—C6	1.5469 (19)		
			
C1—O1—HO1	109.5	C2^ii^—C5—H5	112.5
C6—O2—HO2	109.5	O1—C1—C5	118.51 (12)
C9—C10—C6	95.61 (11)	O1—C1—C2	119.82 (11)
C9—C10—C7^i^	110.40 (11)	C5—C1—C2	97.34 (10)
C6—C10—C7^i^	112.38 (11)	O1—C1—H1	106.7
C9—C10—H10	112.4	C5—C1—H1	106.7
C6—C10—H10	112.4	C2—C1—H1	106.7
C7^i^—C10—H10	112.4	O2—C6—C7	119.86 (11)
C3—C2—C1	95.56 (11)	O2—C6—C10	118.46 (11)
C3—C2—C5^ii^	110.74 (11)	C7—C6—C10	97.50 (10)
C1—C2—C5^ii^	111.67 (11)	O2—C6—H6	106.6
C3—C2—H2	112.6	C7—C6—H6	106.6
C1—C2—H2	112.6	C10—C6—H6	106.6
C5^ii^—C2—H2	112.6	C4—C3—C2	109.17 (12)
C8—C7—C6	95.63 (11)	C4—C3—H3	125.4
C8—C7—C10^i^	110.29 (11)	C2—C3—H3	125.4
C6—C7—C10^i^	111.38 (11)	C9—C8—C7	109.28 (12)
C8—C7—H7	112.8	C9—C8—H8	125.4
C6—C7—H7	112.8	C7—C8—H8	125.4
C10^i^—C7—H7	112.8	C8—C9—C10	109.46 (12)
C4—C5—C1	95.60 (11)	C8—C9—H9	125.3
C4—C5—C2^ii^	110.56 (11)	C10—C9—H9	125.3
C1—C5—C2^ii^	112.16 (11)	C3—C4—C5	109.41 (12)
C4—C5—H5	112.5	C3—C4—H4	125.3
C1—C5—H5	112.5	C5—C4—H4	125.3

Symmetry codes: (i) −*x*+1, −*y*, −*z*; (ii) −*x*+2, −*y*+1, −*z*.

### Hydrogen-bond geometry (Å, °)

**Table:**

*D*—H···*A*	*D*—H	H···*A*	*D*···*A*	*D*—H···*A*
O1—HO1···O2^iii^	0.82	1.95	2.7452 (15)	163
O2—HO2···O1	0.82	1.99	2.8005 (14)	170

Symmetry codes: (iii) *x*, −*y*+1/2, *z*−1/2.
